# Three-Year School-Based Multicomponent Intervention May Change Fruit and Vegetable Preferences in Primary School Children—A Quasi-Randomized Trial

**DOI:** 10.3390/nu15163505

**Published:** 2023-08-08

**Authors:** Ana Ilić, Ivana Rumbak, Ružica Brečić, Irena Colić Barić, Martina Bituh

**Affiliations:** 1 Department of Food Quality Control, Faculty of Food Technology and Biotechnology, University of Zagreb, Pijerottijeva 6, 10000 Zagreb, Croatia; ailic@pbf.hr (A.I.); icolic@pbf.hr (I.C.B.); mbituh@pbf.hr (M.B.); 2 Department of Marketing, Faculty of Economics and Business, University of Zagreb, Trg J.F. Kennedy 6, 10000 Zagreb, Croatia; rbutigan@net.efzg.hr

**Keywords:** childhood, fruit preferences, nutrition intervention, school settings, vegetable preferences

## Abstract

Preference could be the trigger for fruit and vegetable (FV) consumption in children and could be modified by appropriate intervention to increase the acceptance of FVs. The primary objective of this study was to investigate the effects of the three-year school-based multicomponent intervention “Nutri-školica” on the FV preferences of primary school children. It also aimed to explore whether a positive change in FV preferences could lead to an increase in actual FV consumption. The study was conducted in 14 primary schools from the city of Zagreb on 193 children (52.3% boys; age, 7.7 ± 0.4 years; *n* = 85 in the control group and *n* = 108 in the intervention group) who completed a preference questionnaire before and after the intervention with a 5-point hedonic smiley-face scale, where 5 means “I like it a lot.” The per-protocol approach was used for data analysis (28.3% of children from the study sample). After the intervention, children in the intervention group (before: 3.1 ± 0.8; after: 3.5 ± 0.8) increased their FV preferences significantly more than children in the control group (before: 3.2 ± 0.8; after: 3.3 ± 0.7). Children’s FV preferences changed most toward the varieties for which they had the least preferences at the beginning of the study. Participation in the intervention had a stronger effect on changing FV intake than change in FV preferences among primary school children. In summary, the present study highlighted that a targeted intervention can increase children’s FV preferences, but that participation in the intervention is substantial for increasing FV intake.

## 1. Introduction

The global issue of inadequate consumption of fruit and vegetables by children remains a major concern [1,2,3,4]. Especially in the face of increasing childhood obesity [5], it is becoming increasingly important to emphasize the well-known health benefits of adequate consumption of fruit and vegetables [6,7,8].

A number of determinants from the socio-ecological environment could potentially impact the dietary intake of fruit and vegetables in children [9,10,11,12]. According to the available literature, among other determinants, it seems that preferences are the most dominant trigger for fruit and vegetable consumption among children [13,14,15]. In several studies, it has been shown that children with greater fruit and vegetable preferences consumed more fruit and vegetables and their consumption was more varied [16,17,18,19]. In addition, in the study which observed plate waste and children’s preferences for fruit and vegetable dishes from primary schools in Zagreb, the results showed that the children wasted more fruit and vegetable dishes for which they had a lower preference [20]. In light of these findings, various dietary interventions aimed at increasing the fruit and vegetable preferences have been implemented, but the results of these interventions have been inconsistent [21,22,23,24,25,26,27]. The variation in results could be due to the fact that the interventions included a different type and number of components and were not of the same duration.

Considering the results of the study, which show that children in Croatia do not eat enough fruit and vegetables [28], a three-year school-based multicomponent nutritional intervention “Nutri-školica” for primary school children was designed and implemented in 14 schools in the city of Zagreb. The intervention aims to increase children’s fruit and vegetable preferences and intake. The components of the intervention were developed in accordance with the socio-ecological model for children, but also taking into account previous knowledge about the success of interventions and their components in changing children’s fruit and vegetable preferences [21,22,23,24,25,26,27]. So far, it is known that after the implementation of the intervention “Nutri-školica”, children in the intervention group consumed significantly more fruit and vegetables and preferred school meals containing fruit and vegetables than children in the control group [29,30]. However, it remains to be investigated whether children’s fruit and vegetable preferences increased after the intervention.

Therefore, the aim of the present study was to examine the effects of the intervention on primary school children’s preferences for fruits and vegetables. In addition, this study aims to test the hypothesis that increased consumption of fruits and vegetables in primary school children is associated with a positive change in their fruit and vegetable preferences.

## 2. Materials and Methods

### 2.1. Study Design and Settings

This prospective longitudinal intervention study was carried out between the 2018/2019 and 2020/2021 school years as part of the pilot project “School Meals and Fruit and Vegetable Intake in Schools With and Without a Garden” under the Horizon 2020 project “Strengthening European Food Chain Sustainability by Quality and Procurement” (Strength2Food, H2020-SFS-2015-2, contract number 678024). The implementation of the pilot project in the selected primary school was approved by the Ethics Committee of the Institute for Medical Research and Occupational Health (100-21/16-8) and the Croatian Ministry of Science and Education and the Education and Teacher Training Agency (602-01/16-01/00388). All study protocols adhered to the principles of the Declaration of Helsinki and were approved by the Ethics Committee of the School of Medicine, University of Zagreb (380-59-10106-19-11/307).

The selection of children from primary schools in the city of Zagreb was carried out according to the methodology of the project, which is described in detail elsewhere [29,31]. In brief, the 14 schools were selected from a total number of 107 primary state schools in the city of Zagreb. The selection process was as follows: (1) Principals of all schools were approached to determine if their school had a garden, and those with gardens were invited to participate in the study. (2) Of all the schools with gardens, seven principals agreed to participate in the study. (3) For the schools without gardens, a statistical randomization algorithm was used (using C# programming language and Oracle Express database with PL/SQL) to select schools based on three inclusion criteria: (a) schools must not belong to the same district, (b) schools must not have a garden, and (c) schools must agree to participate in the study. To ensure sufficient statistical power (ANOVA ω^2^ ≥ 0.14), a minimum number of seven schools was set. After school selection, parents of first-grade children (*n* = 1039) were informed of the study purpose and protocols during the 2018/2019 school year. Of the total number of first-grade (2018/2019 school year) children from the selected schools (*n* = 1039), for 681 (response rate = 66%), parents gave written consent to participate. In consultation with the principals of each school, the same number or a one-higher number of classes, if an odd number, was assigned in the control group (21 classes, 300 children), while the rest of the classes were assigned in the intervention group (19 classes, 381 children).

Changes in the preferences and consumption of fruit and vegetables were the primary outcomes of the study, measured before and after the three years of intervention and analyzed in comparison with the control group. The secondary outcomes of the study were the associations between children’s fruit and vegetable consumption and preferences and their sociodemographic and lifestyle characteristics. The present study shows the primary and secondary outcomes concerning children’s fruit and vegetable preferences.

### 2.2. Participants

The study participants were first-grade children with a mean age of 7.7 ± 0.4 years at the beginning of the study and 9.8 ± 0.4 years after the intervention. Parents of all children provided the required information by completing an online questionnaire about their children’s date of birth and sex (with the option to select “male” or “female”). Analyses were conducted using the per-protocol approach. Before the intervention, 583 children (85.6% of the total sample) completed the fruit and vegetable preferences questionnaire, and 233 children (34.1% of the sample) did so after the intervention. Finally, 193 children (28.3% of the total sample; 85 in the control group and 108 in the intervention group) who completed the questionnaires on both occasions were included in this study. To compare the pre- and post-intervention changes between the control group and the intervention group with a power of 80% (α = 0.05), at least 128 children had to be included in the study (version 3.1.9.2; Heinrich Heine University Dusseldorf, Dusseldorf, Germany). The hypothesis that positive change in the fruit and vegetable preferences is associated with an increase in fruit and vegetable intake was tested on 154 children (22.6% of the total sample; 70 in the control and 84 in the intervention group) who completed both fruit and vegetable preferences questionnaire and food frequency questionnaire before and after the intervention. At least 55 children were required for these analyses (80% power; α = 0.05) (version 3.1.9.2; Heinrich Heine University Dusseldorf, Dusseldorf, Germany).

### 2.3. Intervention

A three-year multicomponent school-based intervention entitled “Nutri-školica” (translated as Little nutrition school) was implemented in 14 schools into selected classes. The 7-step approach was used for the design and implementation of the intervention [32]. The goals of the intervention were to increase the fruit and vegetable preferences and intake in primary school children.

As mentioned in detail elsewhere [29], the theoretical framework of the intervention was based on three models and theories of eating behavior change (the knowledge–attitude–behavior model, social cognitivist theory, and self-determination theory). To influence all levels of the children’s socio-ecological environment [11,29,33], the intervention consisted of 23 interactive classroom workshops of 45 min duration provided by a nutritionist with additional pedagogical education, 10 cross-curricular interdisciplinary activities provided by teachers, 13 challenges as homework assignments, visual exposure with education posters in classrooms, education for parents through a website, and a change in existing dishes and the implementation of new ones into the school food system. For each activity, fidelity measures were obtained either by the researchers or by the teachers. The fidelity measures encompassed several aspects, such as the count of educational activities conducted, the number of participating children in these activities, the completion rate of challenges, the presence of educational posters in the classroom, the frequency of emails sent to parents with educational blog posts, and a tally of interdisciplinary activities carried out. The 23 interactive classroom workshops were the main component of the intervention focused on postulates of a healthy diet, with a special emphasis on fruit and vegetable consumption. The research team designed all educational materials, including brochures, presentations, posters, and teaching aids. The interactive workshops were conducted during regular school lessons. Because of the COVID-19 pandemic, 19 to 23 workshops were held in intervention classes, and children participated in 15 or more workshops. The teachers conducted up to 4 of 10 cross-curricular interdisciplinary activities. The educational posters (*n* = 5) were hung up in all 19 intervention classes during the scheduled time. The children were sensory-exposed to various fruit and vegetables through 13 challenges as homework assignments, for which they received stickers and prizes. On average, per intervention class, 22% of children completed all 13 challenges, 53% completed approximately 10 to 12 challenges, and 25% completed 6 to 9 challenges. The education of parents was conducted via official websites through blog posts. All parents received email notifications with links to the posts, but we have no information about how many of them read the posts. The educational material was distributed only to the children in the intervention classes, their parents, and their teachers.

### 2.4. Assessment of Fruit and Vegetable Preferences

Children’s fruit and vegetable preferences were rated using a 5-point hedonic smiley-face scale, where a rating of 1 indicated “I dislike it a lot,” and a rating of 5 indicated “I like it a lot.” [34,35,36]. In addition, children could indicate on the questionnaire if they never ate the fruit or vegetables listed in the questionnaire. Children completed preference questionnaires before and after the intervention in the classrooms with the help of the research team and teachers (approximately 30 min). The questionnaire consisted of a list of 26 types of fruit and 28 types of vegetables with names and pictures. The selection of fruit and vegetables in the questionnaire was based on frequency of consumption from available national consumption data [37]. Data analysis was performed on a total of 50 types of fruit and vegetables (excluding potatoes, beans, and chickpeas) according to the World Health Organization definition of fruit and vegetables [38]. Therefore, overall fruit and vegetable preference was presented as mean value of 50 fruit and vegetables. The internal validity of the overall preferences was tested with Cronbach’s alpha before (α = 0.942) and after (α = 0.945) the intervention. In addition, children’s preferences were estimated as fruit preference (a mean value of 26 fruit) and vegetable preference (a mean value of 24 vegetables). The internal validity of the 26 fruit preferences before (α = 0.930) and after (α = 0.940) the intervention and the 28 vegetable preferences before (α = 0.891) and after (α = 0.893) the intervention was tested with Cronbach’s alpha.

### 2.5. Assessment of Fruit and Vegetable Intake

To estimate fruit and vegetable intake in children, the validated semi-quantitative food frequency questionnaire was used [39]. The results of the questionnaire indicate daily fruit and vegetable intake in grams. The questionnaires were completed online by parents together with their children before and after the intervention.

### 2.6. Anthropometric Measurements

The anthropometric assessment included measurements of body weight (±0.1 kg) and height (±0.1 cm). Both were measured by a trained person using a combined medical digital scale and stadiometer (Seca, Type 877-217, Vogel & Halke Gmbh & Co., Hamburg, Germany) during physical education and health classes according to the standard protocols. Data on body mass index (kgm^−2^) were obtained from height and body weight measurements. For each child, sex- and age-standardized z-scores for height, weight, and body mass index were determined with AnthroPlus software version 1.0.4, using the cut-off points suggested by the World Health Organization [40,41].

### 2.7. Physical Activity Level

Physical activity levels were assessed using the Physical Activity Questionnaire for Older Children (PAQ-C), which was accessible online and completed by both parents/caregivers and children [42]. The questionnaire contains 9 questions about the physical activity of children over the last week. The final score (5-point scale) divides children into three categories: insufficiently physically active (1–2 points), moderately physically active (3 points), and very physically active (4–5 points). The questionnaire is validated for the Croatian child population [43].

### 2.8. Sleep and Screen Time

Parents completed a general questionnaire online with 19 questions, of which the questions on sleep and screen time were included in this study. The average daily sleep duration (minutes) was calculated from questions about when their child usually goes to bed and wakes up on school days and on weekends [44,45]. In addition, average daily screen time (minutes) was calculated from questions about the number of hours their child watches TV, plays video games, etc., on school days and weekends [45,46,47].

### 2.9. Statistical Analysis

Data analysis was conducted utilizing IBM SPSS Statistics v. 23.0, released in 2015 (IBM SPSS Statistics for Windows, Armonk, NY, USA: IBM Corp.). Categorical data were reported as frequencies or percentages. The continuous data were reported as means and standard deviations, except children’s liking scores for each fruit and vegetable before and after the intervention (mean and standard error), which were skewed according to the Shapiro–Wilk normality test. Differences in the demographic and anthropometric characteristics and lifestyle habits of children at baseline between the control and the intervention group were tested using Student’s t-test for independent samples. The Pearson correlation coefficient was calculated to estimate the correlations between children’s preferences and their weight status, as well as lifestyle habits, while the point biserial correlation was used to estimate the correlation between children’s preferences and sex. A two-way ANCOVA with repeated measures adjusted for participation in school garden education as a covariate was conducted to compare before- and after-intervention changes in children’s fruit and vegetable preferences and the number of unfamiliar fruit and vegetables in the control and the intervention group. Differences in the distribution of children who were familiar with all fruit and vegetables in the questionnaire before and after the intervention in the control and the intervention group were tested using Fisher’s exact test. The Wilcoxon signed-rank test was used to estimated differences in children’s liking scores for fruit and vegetables before and after the intervention separately in the control and in the intervention group. Multivariable linear regression analysis was carried out to estimate the longitudinal relationship between change in the fruit and vegetable preferences and change in the fruit and vegetable intake. The change in the fruit and vegetable intake was assessed in two models where fruit and vegetable intake after the intervention was the outcome. In model 1, the fruit and vegetable intake at the baseline of the study and change in the fruit and vegetable preference (after the intervention–before the intervention) were the independent variables which contributed to significant variances in the fruit and vegetable intake after the intervention. In model 2, participation in the intervention and in school gardening were added as an independent variable to the ones in model 1. A significance level of *p* < 0.05 was used for all analyses.

## 3. Results

**Figure 1:**
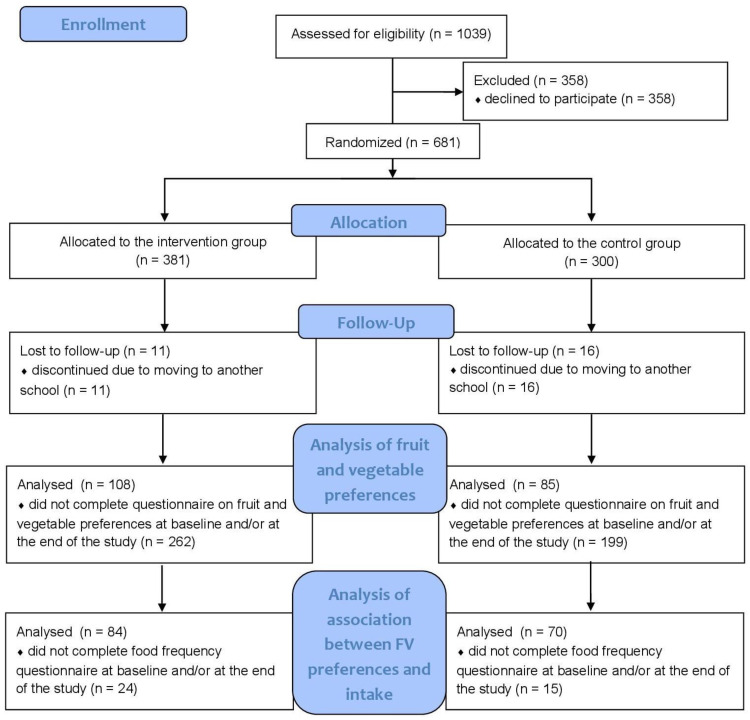
The CONSORT flow diagram for the present study.

As shown in the diagram in Figure 1, out of a total of 681 children, 96% completed the intervention. The reason some dropped out of the study was because they were transferred to another school. Data analyses were performed per protocol; therefore, the analysis included 193 children (85 in the control group and 108 in the intervention group) who completed the preference questionnaire before and after the intervention. In addition, 15 children from the control group and 24 children from the intervention group were dropped from the analysis of the association between change in fruit and vegetable intake and change in preferences because they had not completed both the fruit and vegetable preference questionnaire and the food frequency questionnaire before and after the intervention.

No differences in demographic, anthropometric, and lifestyle characteristics were found between the control and intervention groups at baseline (Table S1) or between the children who dropped out of the study and those who were included in the analysis (Table S2). At baseline, children’s fruit and vegetable preferences were unrelated to sex, weight status, and lifestyle characteristics (Table S3).

As shown in Table 1, the children’s preferences in the intervention group for fruit (before: 3.5 ± 1.1; after: 3.8 ± 0.9; *p* = 0.016), vegetables (before: 2.8 ± 0.9; after: 3.3 ± 0.9; *p* = 0.003), and fruit and vegetables (before: 3.1 ± 0.8; after: 3.5 ± 0.8; *p* = 0.003) increased significantly after the three-year intervention compared to the control group. Before the intervention, fewer than 10% of the children in both groups knew all 50 fruits and vegetables from the preference questionnaire (Table 2). After the intervention, the proportion of children familiar with all fruits and vegetables from the questionnaire increased in both groups, while the number of unknown fruits and vegetables per child decreased. The three most common unknown fruits and vegetables remained the same after the intervention, but the proportion of children who were unfamiliar with the named fruit and vegetables decreased by more than half (Table 2).

The differences in children’s liking scores for fruit and vegetables before and after intervention in the control and the intervention group were presented in Table 3. No differences were found in the liking scores between different fruits before and after the intervention in the control group. In contrast, in the intervention group after the intervention, the preference for bananas significantly decreased (before: 4.4 ± 0.1; after: 4.4 ± 0.1; *p* = 0.028), but the preference for pineapple (before: 3.3 ± 0.2; after: 3.6 ± 0.2; *p* = 0.046), pomegranate (before: 3.0 ± 0.2; after: 3.7 ± 0.2; *p* < 0.001), and currants (before: 2.8 ± 0.2; after: 3.3 ± 0.2; *p* = 0.044) increased. For nine different vegetables, children’s preferences increased in both groups after intervention.

Table 4 shows the results of multivariable linear regression analysis. In model 1, the results showed that fruit and vegetable intake at baseline and change in fruit and vegetable preference significantly predicted fruit and vegetable intake after the intervention (Fruit: F(2,151) = 65.77, *p* < 0.001, R^2^ = 0.466; Vegetable: F(2,151) = 71.19, *p* < 0.001, R^2^ = 0.485; Fruit and vegetable: F(2,151) = 113.10, *p* < 0.001, R^2^ = 0.600). The regression coefficients differed and model fit improved in model 2 (Fruit: F(4,149) = 41.49, *p* < 0.001, R^2^ = 0.527; Vegetable: F(4,149) = 39.79, *p* < 0.001, R^2^ = 0.516; Fruit and vegetable: F(4,149) = 79.49, *p* < 0.001, R^2^ = 0.681) from model 1 after intervention participation, and school gardening was included in the analysis. In addition, the variables included affected the significance of the change in fruit and vegetable preferences, whereas the significance of fruit and vegetable intake at baseline remained the same. Participation in school gardening was not significantly correlated with fruit and vegetable intake in model 2.

## 4. Discussion

This study examined the effectiveness of the three-year school-based multicomponent intervention “Nutri-školica” in changing children’s fruit and vegetable preferences, which is one of the intervention’s primary objectives. To the best of the authors’ knowledge, this is the first evaluated school-based multicomponent nutrition intervention among primary school children in Southeastern Europe. The findings demonstrate the intervention has a positive impact on children’s fruit and vegetable preferences. On a global level, this study shows how a different intervention design can influence children’s fruit and vegetable preferences. In addition, the results highlight the importance of nutritional interventions on changing children’s fruit and vegetable intake.

Different sociodemographic characteristics and lifestyle may influence fruit and vegetable preferences. Therefore, at the beginning of the study, we investigated whether there was a relationship between children’s preferences and sex, weight status, and lifestyle habits. According to the available literature, it had been suggested that sex was strongly correlated with fruit and vegetable preferences, where girls had higher fruit and vegetable preferences than boys [48,49,50]. On the contrary, our results support the findings of other studies that found no association between fruit and vegetable preferences and sex [27,51,52]. In addition, the results do not even suggest that there is a relationship between children’s weight status and their preferences, which supports the existing literature [19,53,54]. The present study adds to the existing literature showing whether there is a relationship between fruit and vegetable preferences and lifestyle habits such as sleep duration, screen time, and physical activity. We hypothesized that children with better lifestyle habits would also have higher fruit and vegetable preferences because they may consume more fruits and vegetables [1,55,56,57,58,59,60,61,62]. However, our results did not confirm this theory. Considering the results obtained, sex, weight status, and lifestyle habits were not used as modifying factors in the evaluation of the intervention. Regarding the other activities of the Strength2Food project and the school selection protocol, the evaluation of the intervention was adjusted for participation in school gardening, as this may influence fruit and vegetable preferences [63,64,65,66,67].

Although fruit and vegetable preferences increased over 3 years in both the control and intervention groups, the three-year school-based multicomponent “Nutri-školica” intervention resulted in a significant increase in preferences in the intervention group compared with the control group. In addition, 70% of students in the intervention group increased preferences for fruit, 81% for vegetables, and 74% for both fruit and vegetables. There was no increase in preference for different types of fruit in the control group after three years, while preference for 11.5% different types of fruit increased in children in the intervention group after the intervention. Preference for 34.6% different types of vegetables increased in children in both groups. It should be noted that the results of the present study indicate that children’s preferences for fruit and vegetables changed most toward the varieties for which students had the least preference at baseline, which is consistent with other studies [68,69]. When looking at the results of this study on preferences for different types of fruit and vegetables, it is noticeable that the children showed a higher preference for sweeter varieties, while they expressed a lower preference for bitter and sour varieties. A previous study found that students aged 7–12 years preferred high-energy foods and fruits, while they disliked bitter, sour, and bland vegetables [70]. This might be due to the fact that young children have a natural aversion to bitter and sour tastes, and preference for these tastes must be learned [13,71]. In general, the reason for the increased preference might be that children had more opportunity to learn about different fruits and vegetables in the third year than at the beginning of the intervention [10,71,72]. It is believed that children may change their preferences if they are exposed to each fruit and vegetable more frequently, and that children need be exposed to each fruit and vegetable at least 15 times to taste it and even more to love it [36,73].

According to the available literature, several interventions have been conducted in the last 10 years in which the effects on changing fruit and vegetable preferences of primary school children were observed [21,22,23,24,25,26,27]. The results of these studies were inconsistent, and in three of them, an increase in preferences was achieved [21,24,26]. In three other studies, preference increased depending on the type of fruit and vegetable [22,23,25], while one study had no effect on fruit and vegetable preference [27]. It is difficult to compare the results of existing studies with each other and with the present study because the interventions differ in their components (e.g., classroom education, cooking classes, taste tests, reinforcement, modeling, etc.), lesson plan, duration, and age of the children involved in the intervention. In addition, the studies differ in the methodology used to determine children’s fruit and vegetable preferences. Namely, children’s fruit and vegetable preferences can be assessed using different hedonic scales, with the most appropriate hedonic scale having five or seven degrees [74]. In the present study, as in all previously mentioned studies [21,22,23,24,25,26,27], the 5-point smiley-face scale was used to assess children’s preferences. However, the questionnaires in the existing literature differ from ours in terms of the types of fruit and vegetables, the number of foods, the calculation of the results (mean or summed value of the foods), and the presentation of the results (fruit, vegetables, or both). Given this, it is difficult to determine whether changes in children’s fruit and vegetable preferences in the present study were small, moderate, or large compared with other studies. In addition, it is difficult to compare children’s preferences for each fruit and vegetable in the present study with others because different fruit and vegetables were included in the preference questionnaires. In addition, in most studies, up to 15 types of fruit and vegetables were included in the preference questionnaires, whereas in the present study, 50 different types of fruit and vegetables were included. For example, in one study, it was observed that children least preferred spinach, leeks, zucchini, beets, and Brussels sprouts [48], while in the present study, spinach was among the most preferred vegetables. According to the available literature, only one study estimated the change in preferences for different fruit and vegetables after the intervention [23]. In that study, children increased their preferences for radish, green beans, and beet, while they remained the same for asparagus, avocado, broccoli, cabbage, carrots, celeriac, corn, grapefruit, lettuce, mushrooms, plums, spinach, sweet potatoes, and tomatoes.

Although it is difficult to distinguish which of the individual components of the intervention was more or less successful in changing preferences, there are some similarities in the present intervention among those who experienced positive changes. The intervention “Nutri-školica” is a theory-based multicomponent intervention based on social cognitive theory, self-determination theory, and the knowledge–attitude–behavior model. Social cognitive theory and knowledge–attitude–behavior theory are the most commonly used theories to explain and predict behavior in nutritional interventions. However, self-determination theory is rarely used in interventions with primary school children according to the available literature [11,75,76,77,78,79,80,81]. The present intervention included activities from different layers of the children’s socio-ecological environment, in contrast to previous studies [21,22,23,24,25,26,27,82,83]. The intervention “Nutri-školica” consisted of 23 classroom educations because it has been shown that interventions that included an educational component were more successful in changing preferences than those that did not [21,22,23,24,25,26,27,82,83]. However, it should be noted that the classroom education and interdisciplinary activities were not fully implemented in schools due to epidemic measures during the COVID-19 pandemic. Furthermore, the available literature showed that the intervention, which included only repeated exposure to messages about fruit and vegetables, behavior modeling, and reinforcement, had no effect on the preferences of children aged 5 to 7 years [27]. Nevertheless, these components were included in the intervention “Nutri-školica” because they appeared to have an impact on children’s preferences along with other components [25]. Cooking classes and/or taste tests seem to play an important role in changing fruit and vegetable preference in the existing literature [21,22,25,26,82]. In the present intervention, it was not possible to conduct these types of activities in schools. Therefore, children were given homework in the form of challenges to cook and taste new fruit and vegetables or dishes that contained fruit and vegetables. In addition, in order to familiarize the children with the new flavors of fruit and vegetables and the different ways of preparing them, it was planned to change the school menu during the intervention, but this activity was postponed due to the COVID-19 pandemic. Parents’ educational level may influence children’s eating behaviors and preferences [10,11]. Therefore, this intervention included a component of parent education through a website, and they were also involved in the intervention through homework challenges along with the children. Only one study that examined the effects of the intervention on students’ fruit and vegetable preference involved parental education [22]. However, the direct effect of this intervention component cannot be assessed because of the influence of the others.

It is well known that children who have a greater preference for fruit and vegetables also have more diverse fruit and vegetable intake [16,17,18,19]. The results of the present study emphasize participation in the intervention rather than a positive change in preference for increasing fruit and vegetable consumption in children. The results of the regression analysis suggest that a positive change in preferences over time may predict higher fruit and vegetable intake among children. However, when we included participation in the intervention along with preference in the regression analysis, only participation in the intervention proved significant for increasing fruit and vegetable consumption.

According to the available literature, the present study is one of the few to examine the effects of the intervention on changing fruit and vegetable preferences in primary school children. The evaluation of the “Nutri-školica” intervention suggests that there is a change in preference shortly after the intervention is implemented. However, in order to assess the sustained change in preference, an evaluation needs to be conducted after a longer period of time. Children could change their preferences for fruits and vegetables as they get older if they are exposed to more different types of fruit and vegetables. Therefore, the effect of the intervention was assessed according to the difference in preferences before and after the intervention between the intervention and control groups, that is, older children. The intervention “Nutri-školica” was not fully implemented due to the COVID-19 pandemic, which could have led to a smaller change in fruit and vegetable preferences.

## 5. Conclusions

The findings of this study suggest that the multicomponent school-based intervention “Nutri-školica” had a positive impact on modifying children’s fruit and vegetable preferences. Such a change in preferences could play a role in influencing fruit and vegetable intake. Nevertheless, it is noteworthy that active participation in the intervention demonstrated a more substantial effect on altering fruit and vegetable consumption among primary school children.

## Figures and Tables

**Table 1:** Comparison of children’s preference as liking scores between the control and the intervention groups before and after the intervention ^1^.

Parameter	Items (*n*)	Before Intervention		After Intervention		*p* Values ^2^
		Control Group (*n* = 85)	Intervention Group (*n* = 108)	Control Group (*n* = 85)	Intervention Group (*n* = 108)	
Fruit	26	3.7 ± 1.0	3.5 ± 1.1	3.8 ± 0.9	3.8 ± 0.9	0.016
Vegetables	24	2.8 ± 0.9	2.8 ± 0.9	3.0 ± 0.8	3.3 ± 0.9	0.003
Fruit and vegetables	50	3.2 ± 0.8	3.1 ± 0.8	3.3 ± 0.7	3.5 ± 0.8	0.003

^1^ Continuous variables are presented as mean and standard deviation. On 5-point hedonic smiley-face scale, 5—"I like it a lot”. ^2^ Differences between groups were tested using two-way ANCOVA with repeated measures adjusted for participation in school garden education (*p* < 0.05).

**Table 2:** Comparison of children’s knowledge and familiarity with fruits and vegetables between the control and intervention groups before and after the intervention ^1^.

Parameter	Before Intervention		After Intervention		*p* Values
	Control Group (*n* = 85)	Intervention Group (*n* = 108)	Control Group (*n* = 85)	Intervention Group (*n* = 108)	
Children who were familiar with all fruit and vegetables (*n*)	3 (3.5%)	7 (6.5%)	58 (68.2%)	43 (39.8%)	0.111 ^2^
Unfamiliar fruit and vegetables (*n*)	9 ± 8	10 ± 8	3 ± 3	3 ± 3	0.199 ^3^
Most unfamiliar fruits and vegetables (% of children)	Fruit: Mango (45.9%) Grapefruit (40.0%) Melon (22.4%) Vegetables: Kohlrabi (72.9%) Asparagus (62.3%) Eggplant (43.5%)	Fruit: Mango (52.8%) Grapefruit (47.2%) Currant (34.3%) Vegetables: Kohlrabi (66.6%) Asparagus (57.4%) Eggplant (49.1%)	Fruit: Mango (22.2%) Grapefruit (21.2%) Currant (12.9%) Vegetables: Asparagus (25.0%) Kohlrabi (17.6%) Celeriac (14.1%)	Fruit: Mango (25.0%) Grapefruit (14.8%) Currant (12.0%) Vegetables: Asparagus (25.0%) Kohlrabi (22.2%) Eggplant (13.8%)	- ^4^

^1^ Continuous variables are presented as mean and standard deviation and categorical ones as number (*n*) or percentage (%). ^2^ Difference was tested using Fisher’s exact test. ^3^ Differences between groups were tested using two-way ANCOVA with repeated measures adjusted for participation in school garden education (*p* < 0.05). ^4^ Statistical analyses were not performed.

**Table 3:** Children’s liking scores for fruit and vegetables before and after intervention ^1^.

Control Group (*n* = 85)				Intervention Group (*n* = 108)			
Food	Before	After	*p* Values ^2^	Food	Before	After	*p* Values ^2^
Fruit							
Apple	4.7 ± 0.1	4.7 ± 0.1	0.889	Apple	4.8 ± 0.1	4.7 ± 0.1	0.233
Strawberry	4.6 ± 0.1	4.5 ± 0.1	0.294	Mandarins	4.6 ± 0.1	4.5 ± 0.1	0.228
Watermelon	4.5 ± 0.1	4.4 ± 0.1	0.093	Banana	4.5 ± 0.1	4.4 ± 0.1	0.028
Mandarins	4.5 ± 0.1	4.4 ± 01	0.402	Watermelon	4.5 ± 0.1	4.4 ± 0.1	0.552
Pear	4.4 ± 0.1	4.4 ± 0.1	0.590	Strawberry	4.5 ± 0.1	4.4 ± 0.1	0.466
Banana	4.4 ± 0.1	4.1 ± 0.1	0.090	Pear	4.3 ± 0.1	4.2 ± 0.1	0.402
Cherry	4.3 ± 0.2	4.4 ± 0.1	0.472	Grapes	4.3 ± 0.1	4.2 ± 0.1	0.787
Grapes	4.2 ± 0.1	4.2 ± 0.1	0.624	Orange	4.2 ± 0.1	4.3 ± 0.1	0.402
Apricot	4.0 ± 0.2	4.2 ± 0.1	0.362	Apricot	4.1 ± 0.2	4.0 ± 0.1	0.347
Orange	4.0 ± 0.2	4.2 ± 0.1	0.348	Cherry	4.1 ± 0.2	3.3 ± 0.2	0.291
Nectarine	3.8 ± 0.2	4.1 ± 0.1	0.120	Raspberries	4.0 ± 0.2	4.1 ± 0.1	0.903
Peach	3.8 ± 0.2	4.2 ± 0.1	0.224	Peach	3.9 ± 0.2	4.0 ± 0.1	0.728
Blueberries	3.7 ± 0.2	4.0 ± 0.1	0.128	Lemon	3.8 ± 0.2	3.6 ± 0.1	0.178
Raspberries	3.7 ± 0.2	4.0 ± 0.2	0.245	Plum	3.8 ± 0.2	3.7 ± 0.1	0.688
Lemon	3.7 ± 0.2	3.4 ± 0.2	0.150	Blueberries	3.7 ± 0.2	4.0 ± 0.1	0.100
Plum	3.6 ± 0.2	3.6 ± 0.2	0.845	Nectarine	3.7 ± 0.2	4.0 ± 0.1	0.061
Pomegranate	3.6 ± 0.2	3.5 ± 0.2	0.585	Sour cherry	3.6 ± 0.2	3.3 ± 0.2	0.171
Blackberries	3.6 ± 0.2	2.9 ± 0.2	0.527	Kiwi	3.6 ± 0.2	3.6 ± 0.2	0.954
Sour cherry	3.4 ± 0.2	3.2 ± 0.2	0.297	Blackberries	3.6 ± 0.2	3.3 ± 0.2	0.560
Kiwi	3.4 ± 0.2	3.4 ± 0.2	0.918	Pineapple	3.3 ± 0.2	3.6 ± 0.2	0.046
Pineapple	3.4 ± 0.2	3.3 ± 0.2	0.621	Pomegranate	3.0 ± 0.2	3.7 ± 0.2	<0.001
Currant	3.1 ± 0.2	2.9 ± 0.2	0.475	Fig	2.9 ± 0.2	2.7 ± 0.2	0.145
Melon	2.7 ± 0.2	2.4 ± 0.2	0.181	Currant	2.8 ± 0.2	3.3 ± 0.2	0.044
Fig	2.6 ± 0.2	2.3 ± 0.2	0.298	Melon	2.5 ± 0.2	2.6 ± 0.2	0.471
Grapefruit	2.2 ± 0.2	1.8 ± 0.2	0.034	Grapefruit	2.0 ± 0.2	2.0 ± 0.1	0.905
Mango	2.1 ± 0.2	2.1 ± 0.2	0.951	Mango	2.0 ± 0.2	2.0 ± 0.2	0.918
Vegetables							
Carrot	4.2 ± 0.1	4.3 ± 0.1	0.690	Carrot	4.4 ± 0.1	4.2 ± 0.1	0.240
Cucumber	3.7 ± 0.2	3.8 ± 0.2	0.562	Lettuce	4.0 ± 0.1	4.0 ± 0.1	0.918
Lettuce	3.6 ± 0.2	3.8 ± 0.2	0.278	Cucumber	4.0 ± 0.2	4.0 ± 0.1	0.978
Spinach	3.6 ± 0.2	3.6 ± 0.1	0.610	Peas	3.7 ± 0.1	3.7 ± 0.1	0.599
Green beans	3.6 ± 0.2	3.7 ± 0.1	0.498	Beetroot	3.7 ± 0.2	3.8 ± 0.1	0.806
Cabbage	3.5 ± 0.2	3.7 ± 0.1	0.160	Spinach	3.6 ± 0.2	3.8 ± 0.1	0.439
Peas	3.4 ± 0.2	3.3 ± 0.2	0.724	Cabbage	3.5 ± 0.2	3.8 ± 0.1	0.161
Beetroot	3.4 ± 0.2	3.4 ± 0.2	0.796	Green beans	3.5 ± 0.2	3.8 ± 0.1	0.097
Tomatoes	3.3 ± 0.2	3.4 ± 0.2	0.572	Tomatoes	3.5 ± 0.2	3.6 ± 0.2	0.639
Broccoli	3.2 ± 0.2	3.2 ± 0.2	0.662	Paprika	3.3 ± 0.2	3.3 ± 0.2	0.892
Kale	3.0 ± 0.2	3.0 ± 0.2	0.917	Onion	2.9 ± 0.2	3.2 ± 0.2	0.132
Paprika	2.7 ± 0.2	2.8 ± 0.2	0.357	Broccoli	2.7 ± 0.2	3.0 ± 0.1	0.399
Onion	2.6 ± 0.2	3.1 ± 0.2	0.006	Kale	2.7 ± 0.2	3.3 ± 0.1	0.002
Leek	2.5 ± 0.2	3.0 ± 0.2	0.005	Leek	2.6 ± 0.2	3.1 ± 0.2	0.034
Cauliflower	2.4 ± 0.2	3.1 ± 0.2	0.004	Cauliflower	2.6 ± 0.2	2.9 ± 0.1	0.183
Mushrooms	2.3 ± 0.2	2.6 ± 0.2	0.101	Mushrooms	2.5 ± 0.2	2.6 ± 0.2	0.421
Pumpkin	2.2 ± 0.2	3.2 ± 0.2	<0.001	Zucchini	2.1 ± 0.2	3.1 ± 0.1	<0.001
Zucchini	2.1 ± 0.2	3.0 ± 0.1	<0.001	Celeriac	2.1 ± 0.2	2.1 ± 0.1	0.777
Celeriac	1.9 ± 0.2	2.0 ± 0.2	0.664	Pumpkin	2.0 ± 0.2	2.8 ± 0.2	<0.001
Radish	1.8 ± 0.2	2.2 ± 0.2	0.047	Radish	2.0 ± 0.2	2.5 ± 0.2	0.017
Brussel sprouts	1.5 ± 0.2	1.8 ± 0.1	0.133	Brussel sprouts	1.7 ± 0.2	1.9 ± 0.1	0.289
Eggplant	1.4 ± 0.2	1.9 ± 0.1	0.015	Asparagus	1.3 ± 0.2	1.8 ± 0.2	0.021
Asparagus	1.4 ± 0.2	1.5 ± 0.2	0.272	Eggplant	1.3 ± 0.2	2.1 ± 0.2	<0.001
Kohlrabi	0.8 ± 0.2	1.9 ± 0.2	<0.001	Kohlrabi	1.0 ± 0.2	2.0 ± 0.2	<0.001

^1^ Continuous variables are presented as mean and standard error. On 5-point hedonic smiley-face scale, 5—"I like it a lot”. ^2^ Differences were tested using Wilcoxon signed-rank test (*p* < 0.05).

**Table 4:** Results of the multivariate linear regression analyses with the fruit and vegetable intake after the intervention as the outcome in children (*n* = 154) ^1^.

Variable	Fruit Intake		Vegetable Intake		Fruit and Vegetable Intake	
	β (95% CI)	*p* Values	β (95% CI)	*p* Values	β (95% CI)	*p* Values
Model 1:						
Change in preference	26.165 (0.55–51.78)	0.045	13.07 (0.56–25.85)	0.041	34.08 (2.13–66.03)	0.037
Intake at the baseline	0.785 (0.65–0.92)	<0.001	0.74 (0.61–0.88)	<0.001	0.83 (0.72–0.95)	<0.001
Model 2:						
Change in preference	16.44 (−8.28–41.17)	0.191	8.64 (−3.88–21.17)	0.175	13.14 (−16.39–42.69)	0.381
Intake at the baseline	0.79 (0.66–0.93)	<0.001	0.76 (0.63–0.89)	<0.001	0.857 (0.754–0.960)	<0.001
Intervention	−85.45 (−123.91–−46.99)	<0.001	−27.26 (−44.79–−9.73)	0.003	−117.73 (−155.57–−79.89)	<0.001
School gardening	8.84 (−28.93–46.62)	0.644	0.734 (−16.31–17.78)	0.932	10.00 (−26.75–46.75)	0.592

^1^ Unstandardized regression coefficients β and 95% confidence intervals are presented (*p* < 0.05). Model 1: adjusted for change in preference and intake at baseline. Model 2: adjusted for change in preference, intake at baseline, participation in intervention and participation in school garden education.

## Data Availability

The data are available upon request from A.I.

## Supplementary Materials

The following supporting information can be downloaded at: https://www.mdpi.com/article/10.3390/nu15163505/s1, Table S1: Baseline characteristics of study sample; Table S2: Differences between the characteristics of the non-participants and the participants in the intervention and control groups at baseline; Table S3: Correlation between children’s preferences and characteristics at baseline (*n* = 193).
