# Supplementary material for: Three-Year School-Based Multicomponent Intervention May Change Fruit and Vegetable Preferences in Primary School Children—A Quasi-Randomized Trial
Source: Nutrients. 2023 Aug 8;15(16):3505. doi: 10.3390/nu15163505 (PMC10457900; doi:10.3390/nu15163505)
Supplement: Supplementary file 1 [file nutrients-15-03505-s001.zip › Supplementary Materials_Table S1.pdf]

Table S1. Baseline characteristics of study sample. [1]

Parameter	Total sample (n = 193)	Control (n =85)	Intervention (n = 108)	P values [2]
Age (yr.)	7.7 ± 0.4	7.7 ± 0.3	7.7 ± 0.4	0.376
Boys (%)	52.3	52.9	51.9	0.880
Girls (%)	47.7	47.1	48.1	
Body height (cm)	135.3 ± 5.8	136.0 ± 6.3	134.8 ± 5.3	0.301
Body height-for-age z-score	0.9 ± 0.9	0.98 ± 1.06	0.8 ± 0.8	0.416
Body weight (kg)	31.4 ± 6.7	31.0 ± 6.1	31.8 ± 7.2	0.549
Body weight-for-age z-score	0.8 ± 1.1	0.7 ± 1.0	0.9 ± 1.1	0.331
Body mass index (kgm-2)	17.0 ± 2.7	16.6 ± 2.3	17.4 ± 2.9	0.069
Body mass index-for-age z-score	0.4 ± 1.2	0.7 ± 1.0	0.6 ± 1.2	0.067
Physical activity level	3.1 ± 0.6	3.1 ± 0.6	3.0 ± 0.6	0.504
Sleep time (min/day)	586.8 ± 32.0	586.3 ± 33.4	587.2 ± 31.1	0.686
Screen time (min/day)	133.0 ± 67.2	129.7 ± 68.5	135.7 ± 66.4	0.352

[1] Continuous variables are presented as mean and standard deviation and categorical as percent-age. [2] Differences between groups were tested using Student's t-test ($p < 0.05$).
