# Supplementary material for: Three-Year School-Based Multicomponent Intervention May Change Fruit and Vegetable Preferences in Primary School Children—A Quasi-Randomized Trial
Source: Nutrients. 2023 Aug 8;15(16):3505. doi: 10.3390/nu15163505 (PMC10457900; doi:10.3390/nu15163505)
Supplement: Supplementary file 1 [file nutrients-15-03505-s001.zip › Supplementary Materials_Table S2.pdf]

Table S2. Differences between the characteristics of the non-participants and the participants in the intervention and control groups at baseline [1].

Parameter	Control group (n=300)			Intervention group (n=381)		
	Non-completers	Completers	p Value [2]	Non-completers	Completers	p Value [2]
**Participants (n)**	184	116	-[3]	238	143	-[3]
**Age (yr.)**	7.7 ± 0.3	7.7 ± 0.3	0.795	8.6 ± 0.3	7.7 ± 0.4	0,251
**Sex (%):**						
Boys	51.6	49.1	0.812	48.9	50.3	0.791
Girls	48.4	50.9		51.1	49.7	
**Body height (cm)**	134.8 ± 6.0	135.4 ± 6.2	0.322	135.5 ± 5.7	135.5 ± 5.7	0,949
**Body height-for-age z-score**	0.8 ± 1.0	0.9 ± 1.1	0.157	0.9 ± 0.9	0.9 ± 0.9	0,776
**Body weight (kg)**	31.2 ± 7.2	30.7 ± 5.9	0.550	31.7 ± 6.6	32.2 ± 7.0	0.432
**Body weight-for-age z-score**	0.7 ± 1.1	0.7 ± 1.1	0.918	0.9 ± 1.1	0.9 ± 1.1	0.481
**Body mass index (kgm^{-2})**	17.0 ± 2.8	16.6 ± 2.3	0.159	17.1 ± 2.8	17.4 ± 2.9	0.373
**Body mass index-for-age z-score**	0.4 ± 1.2	0.2 ± 1.1	0.217	0.4 ± 1.2	0.6 ± 1.2	0.323
**Physical activity level**	3.0 ± 0.5	3.1 ± 0.7	0.986	3.0 ± 0.5	3.1 ± 0.6	0,923
**Sleep time (min/day)**	585.4 ± 23.9	580.9 ± 34.2	0.599	590.3 ± 33.5	589.1 ± 32.2	0,897
**Screen time (min/day)**	120.5 ± 50.8	141.8 ± 76.8	0.325	146.8 ± 82.1	134.3 ± 63.2	0.913
**Household income (%):**						
< 5000.00 kn (664.92 €)	0.0	3.4		8.0	1.0	
5000,00 kn – 8000,00 kn (664.93 – 1063.87 €)	17.2	5.7		8.0	7.3	
8000,01 kn – 14000,00 kn (1063.88 – 1861.77 €)	31.0	34.5	0,351	32.0	35.4	0.420
14000,01 kn – 18000,00 kn (1861.78 – 2393.70 €)	24.2	19.5		24.0	24.0	
≥ 18000,01 kn (≥ 2393.71 €)	27.6	36.8		28.0	32.3	

[1] Continuous variables are presented as mean ± standard deviation, while categorical as number or percentages. [2] Differences between groups were tested using Student's t-test for continuous parametric variables, Mann-Whitney U test for continuous non-parametric variables and Chi-square test for categorical variables ($p < 0.05$). [3] Statistical analyzes was not performed.
