# Supplementary material for: Three-Year School-Based Multicomponent Intervention May Change Fruit and Vegetable Preferences in Primary School Children—A Quasi-Randomized Trial
Source: Nutrients. 2023 Aug 8;15(16):3505. doi: 10.3390/nu15163505 (PMC10457900; doi:10.3390/nu15163505)
Supplement: Supplementary file 1 [file nutrients-15-03505-s001.zip › Supplementary Materials_Table S3.pdf]

Table S3. Correlation between children's preferences and characteristics at baseline (n=193).

Parameter	Fruit preference		Vegetable preference		Fruit and vegetable preferences	
	r coefficient	P values	r coefficient	P values	r coefficient	P values
Sex [1]	0.123	0.087	-0.036	0.616	0.057	0.432
Body mass index-for-age z-score [2]	-0.089	0.221	-0.042	0.566	-0.075	0.303
Physical activity level [2]	0.018	0.810	-0.058	0.451	-0.023	0.766
Sleep time (min/day) [2]	-0.016	0.825	-0.004	0.957	-0.012	0.872
Screen time (min/day) [2]	0.037	0.618	0.071	0.341	0.061	0.415

[1] Point-biserial correlation ($p < 0.05$). [2] Pearson correlation ($p < 0.05$).
